# The Influence of Pregnancy and Lactation on Maternal Bone Health: A Systematic Review

**Published:** 2014-12

**Authors:** Pooneh Salari, Mohammad Abdollahi

**Affiliations:** 1 Medical Ethics and History of Medicine Research Center, Tehran University of Medical Sciences, Tehran, Iran; 2 Faculty of Pharmacy, Tehran University of Medical Sciences, Tehran, Iran; 3 Pharmaceutical Sciences Research Center, Tehran University of Medical Sciences, Tehran, Iran; 4 Endocrinology & Metabolism Research Center, Tehran University of Medical Sciences, Tehran, Iran

**Keywords:** bone loss, pregnancy, lactation, parity

## Abstract

Osteoporosis is considered as an important public health problem especially in postmenopausal women. There are some hypotheses support the contributory effect of pregnancy and lactation on osteoporosis later in life. High calcium demand during pregnancy and lactation and low estrogenic state support those hypotheses. Numerous studies have investigated on the issue but there is no consensus about the contributory effect of pregnancy and lactation on osteoporosis. To explore the current state of fact, in the present study, all bibliographic databases were searched and all relevant studies on the topic of osteoporosis, lactation, and pregnancy were reviewed.

The review shows that despite of controversial results, pregnancy may have protective effect on bone especially if followed by lactation.

## Introduction

Osteoporosis is a chronic metabolic bone disease developing in both genders, but osteoporosis in postmenopausal women is of higher importance and is considered as a public health problem. Investigations try to better understand pathogenesis of osteoporosis, bone metabolism and the role of inflammatory pathways as well as the link with chronic senile diseases to find superior ways of prevention or treatment (1-5). Several contributory factors are considered essential in regulating bone metabolism as well as reaching peak bone mass in young ages (6-11). Both pregnancy and lactation are physiologic conditions mostly occurring in young women aged ≤ 40 years, in which calcium homeostasis is high. Pregnancy and lactation are proposed as two risk factors for postmenopausal osteoporosis but the studies do not support the hypothesis. Studies show 4-6% bone loss during the first six months of lactation because of hypoestrogenic state and calcium loss in breast milk (12). However there is no consensus about bone loss during lactation or the long-term effects of pregnancy and lactation on bone.

High calcium demand during pregnancy and lactation make women more prone to bone resorption and subsequent osteoporosis. Although hormonal changes cause calcium loss and result in increased bone resorption, bone resorption may be reversed after delivery (13, 14). Therefore, pregnancy and lactation can have dual effect on bone; beneficial or detrimental. The final net effect of pregnancy and lactation on bone is not obviously known and there is no consensus on the issue. In this article we reviewed and criticized the most relevant studies evaluated the impact of pregnancy and lactation on bone to provide most acceptable opinion.

***Data sources***

PubMed, Web of Science (ISI) and Scopus were looked for by keywords pregnancy, lactation and bone with no time limitation. We limited our search to original English papers only. All relevant papers were reviewed and data extracted.

Bone metabolism during pregnancy and lactation

Calcium homeostasis is significantly altered during pregnancy and lactation. In pregnancy 2-3% of maternal calcium is transferred to fetus mostly in the second and third trimester (15). During lactation 300-400 mg calcium per day is transferred into breast milk (16). Accordingly, many regulatory mechanisms such as renal calcium reservation, intestinal absorption, and bone resorption are stimulated (17). Because of high calcium demand during pregnancy, the rate of intestinal calcium reabsorption and bone turnover are increased (18). In the lactation period, calcium is preserved by kidneys to maintain bone metabolism (19). Despite of the involvement of several counter-regulatory pathways during pregnancy, bone mineral density decreases about 3%. The bone loss is counterbalanced by higher circulating levels of dihydroxyvitamin D, changes in parathyroid hormone (PTH), growth hormone, prolactin, estrogen, nutritional habits, body weight, and lifestyle (20,21). Little is known about the regulatory mechanism of calcium metabolism during lactation, but it is mostly mediated by PTH-related peptide (PTHrP) and hypoestrogenic state (16).

During pregnancy PTHrP is secreted from maternal and fetus tissues which increases dihydroxyvitamin D3, suppresses PTH, controls placental calcium transport and protects maternal skeleton (22). 

Furthermore, during pregnancy and subsequent lactation, the ovarian activity is low (23). During lactation, increased estrogen level may equalize the imbalance between bone resorption and bone formation (24). Estrogen deficiency during postpartum amenorrhea causes bone loss and a positive association between serum estradiol and postpartum bone mineral density (BMD) was determined (25) but the importance of estrogen is not fully understood. Meanwhile menses resumption has been proposed as a major modulator of bone metabolism after pregnancy and lactation (26). 

Taken together, high calcium demand and estrogen deficiency stimulate bone metabolism during pregnancy and lactation.

Pregnancy, lactation and bone

Available clinical and epidemiological data do not support permanent bone loss during pregnancy and lactation (27) and there is no consensus on the long-term effect of pregnancy and lactation on bone mass. What is the blind spot is the longitudinal effects of pregnancy and lactation on BMD and prevalence of osteoporosis. Because of a potent correlation between lactation and pregnancy, both are considered as a combined risk factor. 

BMD changes in pregnancy and lactation

Pearson et al showed non-significant 1% decline in BMD at spine and hip during pregnancy and a constant pattern of bone loss during lactation especially at spine. In addition they reported restoration of 5% of the preconceptual BMD value at spine and trochanter with less recovery at total hip (28). Kolle et al observed the association of low BMD and previous pregnancy in Norwegian premenopausal women (29). In a cohort of healthy postpartum women Holmberg-Marttila et al determined systematic site specific pattern of bone loss during postpartum amenorrhea (PPA) and bone restoration after menses resumption affecting by lactation habits (30). Details of studies are summarized in Table 1.

Parity and bone

Several investigations showed long-term supportive effect of parity on bone (20,31). To & Wong observed less BMD decrement in multiparous women compared with primiparous (32). 

Their results were confirmed in pre- and post-menopausal women (33,34). In contrast some researchers found high parity as a risk factor for osteoporosis (35) and indicated that having 6 children or more is associated with low spinal and hip BMD in postmenopausal women (36). Parra-Cabrera et al retrospectively assessed the effect of pregnancy on BMD in women aged (26-73 years) and reported detrimental effects of the number of pregnancies on BMD (37). Several other studies could not show the association between bone density and parity or lactation even in long term (38-41). 

Some studies reported a weak to moderate protective effect of parity on risk of fracture (42, 43) while the results of the study of Parazzini et al. are against it (44). Details of the studies appeared in Table 2.

**Table 1:** Studies which show the impact of pregnancy on bone

**Author **	**Type of study**	**Subjects**	**Conclusion**
Alderman et al. 1986	Case-control	917 PoM	RF in multiparous women (≥4 birth)= RF women without birth; RF in breast-fed >2yrs= RF in women without breast feeding
Pearson et al. 2004	Longitudinal	60 PrM	Constant bone loss during pregnancy; most of them returned to within 5% of normal BMD
Kolle et al. 2005	Cohort	145 (13-39 yrs)	Association of low BMD and pregnancy (95%CI -0.081- -0.015, B = -0.048) (P = 0.005)
Holmberg-Marttilla et al. 2000	Cohort	41 postpartum women	Systematic bone loss during PPA [at lumbar spine, mean -2.2%;95% CI, -3.4%- -0.9%; P<0.01)], [at femoral neck mean, -3.6%; 95% CI, 4.5% - -2.6%; P< 0.0001)]; BMD recovery after menses resumption [lumbar spine, mean, 3.3%; 95% CI,2.0%-- 4.6%, P < 0.0001)], [femoral neck mean, -1.0%; 95% CI,-1.7%- -0.2%; P = 0.02)]

RF = risk factor; PrM = premenopausal women; PoM = postmenopausal women; BMD = bone mineral density; yrs= years

PPA = postpartum amenorrhea; B = regression coefficient

**Table 2:** Studies which show the impact of parity on bone

**Author **	**Type of study**	**Subjects**	**Conclusion**
Murphy et al. 1994	Retrospective	825 (41-76 yrs)	Parity is a significant independent predictor of BMD; 1% increase in BMD per live birth
Hoffman et al. 1993	Case-control	348 (≥45 yrs)	Lactation is not associated with hip fracture (OR, 0.8; 95% CI, 0.42-1.55)
Tuppurainen et al. 1995	-	1605 PrM & PoM	The significant positive association between parity and BMD, Higher BMD in parrous postmenopausal women
Fox et al 1993		2230 PoM	The significant positive association between parity and BMD
Berehi et al. 1996	Open study	159 Omani women (20-70 yrs)	Multiparity does not influence lumbar spine BMD
Cummings et al 1995	Cohort	9516 PoM	Lactation (OR, 0.9; 95% CI 0.8-1.0) is not associated with risk of hip fracture
O´Neil et al 1997	Cross sectional	7530 PoM	Parity & lactation does not affect risk of vertebral deformity
Melton et al. 1993	Cross-sectional	304 PoM	Pregnancy & lactation have little long term effect on bone mass
Streeten et al 2005	Observational	424 (≥40 yrs)	Parity is strongly associated with BMD in women aged 50-59 yrs
Hillier et al 2003	Prospective	9704 PoM	↑Parity →↓ HF (HR, 0.87; 95% CI, 0.81-0.94)
Petersen et al 2002	Cross-sectional & prospective	5102 PoM	Pregnancy is associated with low RF[number of births vs number of HF (OR, 1.22; 95% CI, 0.56-2.65)
Parazzini et al 1996	Case-control	796 PoM	No influence of reproductive factors on RF[RF in parous vs nulliparous women (OR, 0.8; 95% CI, 0.6-1.3)
Demir et al. 2008	Cohort	2769 PoM	High parity is a risk factor for low BMD (OR, 1.14; 95% CI, 1.08-1.21)
Allali et al 2007	Cross-sectional	730 PoM	↑ number of pregnancies →↓ hip & spine BMD; no correlation between parity and peripheral fractures
Parra-Cabrera et al. 1996		313 (26-83 yrs)	Number of pregnancies → deleterious effect on BMD (r = -0.013, P = 0.007)

RF= risk factor; PrM= premenopausal women; PoM= postmenopausal women; BMD= bone mineral density; yrs= years

PPA= postpartum amenorrhea; OR= odds ratio; CI= confidence interval; HF= hip fracture; HR= hazard ratio

Lactation and bone

It was reported that bone metabolism is higher in lactating mothers with longer period of breastfeeding than that of non-lactating mothers (45). It is hypothesized that after discontinuing breast feeding, bone resorption returns to normal while bone formation continues (46). During lactation, 4-7% bone loss occurs in lumbar spine and femoral neck which is reversed about one year after weaning in a site specific manner (47, 48). Therefore bone loss during lactation seems to be partial (49) and there is the possibility of complete restoration of bone density (50,51). 

Although some former studies insisted on the protective effect of lactation on BMD (52, 53), new studies indicate detrimental effects (54,55) while some others showed no significant impact (56). In addition there are some reports of negative relationship between duration of lactation and BMD (57-59) and some reports of no relationship (60). Likewise, the results of such studies in Japan, America and Sri Lanka showed opposite results (61, 62). 

More et al measured BMD in pregnant women and observed that bone mass recovery continues until 12 months postpartum in women with less than one month breastfeeding. In mothers with up to 6 months breastfeeding, bone loss stops 6 months after delivery and 6 months later, it reaches baseline level. In addition, they found that if lactation continues for 12 months, bone mass does not reach baseline level (63).

Shilbayeh indicated lactation, its frequency (4 times or more) and duration (1-6 months) as significant protective elements of bone density (64). Dursun et al introduced total duration of breast feeding as an important predictor of lumbar spine BMD and observed significant lower BMD at spine and femur in Turkish women with longer duration of lactation (54). In contrast, Aksakal et al determined no significant effect of lactation on bone in pre- and post-menopausal Turkish women (65). 

In a recent cohort study, Khoo et al indicated that duration of lactation is a negative predictor of BMD at hip and spine (58) which is in agreement with the study of Rojano-Mejia et al in Mexican women (59). 

In a recent study, Wiklund et al displayed the protective effect of lactation on bone size and strength in direct correlation with its duration (66) while Yazici et al found no effect of lactation and its duration on postmenopausal women (67). Details of studies are summarized in Table 3.

**Table 3:** Studies which show the impact of lactation on bone

**Author **	**Type of study**	**Subjects**	**Conclusion**
Holmberg-Marttila et al. 2003	Cohort	32 healthy mothers after delivery	Rate of bone formation is higher in mothers with longer period of breastfeeding
Polatti et al. 1999	Cohort	308 lactating mothers	Lactation→↓BMD→recovery after weaning
Affinito et al 1996		36 (24-31 yrs)	Significant decrease in BMD during lactation and partial recovery 6 months after weaning
Shilbayeh 2003	Cross-sectional	400 (19-85 yrs)	Lactation, its frequency (≥4) (OR, 0.16; 95% CI, 0.03-0.84) and interval (1-6 months) are bone protective (OR, 0.07; 95% CI, 0.006-0.85)
Hansen et al. 1991	Longitudinal	121 PoM	Lactation is bone protective
Feldblum et al. 1922	Cross-sectional	352 PrM	Lactation has beneficial effect on BMD
Hu et al. 1994	Cross-sectional	775 (35-75 yrs)	Significant positive association between lactation and BMD
Dursun et al. 2006	Cross-sectional	1486 PoM	Significant negative association between total duration of lactation and BMD
Gur et al. 2003		509 PoM	Extended lactation leads to lower BMD
Kalkwarf et al 1995	Cohort	113 postpartum	Bone loss during lactation will be restored after weaning
Chowdhury et al. 2002	Cross-sectional	400 (20-81 yrs)	Negative correlation between duration of lactation and BMD
Carranza-Lira et al. 2002		50 (35-40 yrs)	Number of pregnancy and duration of lactation do not affect BMD
More et al. 2001	Prospective	38 pregnant women	Significant correlation of duration of lactation with bone loss (r = -0.729)
Aksakal et al 2008	-	78 PrM & 18 PoM	No significant correlation between lactation period and BMD
Chantry et al. 2004	Cross-sectional	819 (20-25 yrs)	Lactation (mean, 0.049; 95% CI, 0.002-0.095) is associated with higher BMD in adolescent mothers
Khoo et al. 2011	Cohort	2000 (65-98 yrs)	Lactation period is significant negative predictor of BMD (OR, -0.4; 95% CI -0.6 to -0.2)
Rojano-Mejia et al. 2011	-	567 PoM	Lactation period is a risk factor for osteoporosis (OR , 2.48; 95% CI, 1.41-4.38)
Kojima et al 2002	Cross-sectional	456 PrM & 713 PoM	Inverse correlation of lactation period with BMD in PrM (95% CI, -0.464- -0.098); no significant correlation in PoM
Lenora et al. 2009	Cross-sectional	210 (46-98 yrs)	No detrimental effect of parity (95% CI, 6.4-7.2) and lactation (95% CI, 130.8-141.5)on BMD in PoM
Wiklund et al. 2012	Retrospective	145 (16-20 yrs)	Lactation is beneficial to bone strength
Yazici et al. 2011	Retrospective	586 PoM	Lactation has no effect on BMD of PoM and lactation period is not a risk factor for low BMD (OR, 0.999; 95% CI 0.993-1.005)
Berning et al 1993	Cross-sectional	94 PoM	Total lactation (r = 0.29, P = 0.005) period rather than parity (r = 0.26, P = 0.01)is associated with BMD
Cure-Cure et al 2002	-	1855 PoM	Osteopenia is higher in nulliparous women (OR, 2.01; 95% CI, 1.2-3.35), Parity is a protective factor against osteoporosis
Lissner et al 1991		126 Prm & PoM	Higher parity & Total duration of lactation was associated with low BMC
Henderson et al. 2000	Cohort	30 grand multiparous	Repeated pregnancy & lactation does not affect BMD
Grainge et al. 2001	Cross-sectional	580 PoM	Number of pregnancies were strongly associated with increased BMD; no association between lactation and BMD was found
Zhang et al. 2003	Cross-sectional	214 PoM, 428 PrM	More parity →negative effect on BMD, no significant impact of lactation on BMD
Paton et al. 2003	Retrospective	1935 (≥18 yrs)	No long term detrimental effect of pregnancy or lactation on BMD
Hill et al. 2008	Cohort	340 PoM	History of breast feeding was associated with higher BMD (OR, 1.06, 95% CI, 0.11-2.01)
Schnatz et al. 2010	Retrospective, prospective	619 (≥49 yrs)	Multi parity (OR, 0.45; 95% CI, 0.22-0.95) & lactation (OR, 0.38; 95% CI, 0.2-0.72)→↓ chance of osteoporosis
Hadji et al. 2002	Cohort	2080 PoM	No association between parity or lactation and ultrasonometry variables was found

RF = risk factor; PrM = premenopausal women; PoM = postmenopausal women; BMD = bone mineral density; yrs = years

PPA = postpartum amenorrhea; BMC= bone mineral content; OR = odds ratio; CI = confidence interval

Parity and lactation

The protective effects of previous lactation history and parity on bone were demonstrated in some studies (55, 68, 69) hence there is no consensus. Some former studies stated protective effect of parity and duration of lactation on BMD (70); some mentioned a negative association (71) and the others found no association (72-74). Kojima et al investigated the effect of parity and lactation on BMD in pre- and post-menopausal women in a cross-sectional study. They stated an inverse correlation between total lactation period and BMD in premenopausal women but found no association between them in the postmenopausal women and they concluded that lactation and parity does not have major effect on BMD later in life (75). 

Zhang et al confirmed the detrimental effect of parity on BMD with no influence of lactation in postmenopausal Chinese women while in premenopausal women none of them caused significant association (76). Karlsson et al studied the effect of pregnancy and lactation in 73 women aged 20-44 years and observed significant decrease in spine and body BMD after delivery. In the first 12 months after delivery, the BMD of non-lactating mothers did not significantly change however 12 months after delivery, lumbar spine BMD showed significant increment (77). Meanwhile higher BMD loss was seen in lactating mothers. They could not find correlation between parity and BMD (77).

Hill et al reported the association of >5% increase in BMD of African Caribbean women with parity and lactation in age-adjusted models but the correlation was not significant (78). Lenora et al conducted a cross sectional study in Sri Lankan women and found no detrimental effect of parity and duration of lactation on BMD in postmenopausal women (62). In another former study Chantry et al indicated the positive association between lactation, age of pregnancy and bone (79). 

In addition, ultrasonometry of the heel showed no significant association between ultrasonometry variables and parity or lactation in 2080 postmenopausal women (80). Details are summarized in Table 4.

**Table 4:** Studies which show the impact of parity and lactation on bone

**Author **	**Type of study**	**Subjects**	**Conclusion**
Paganini-Hill et al. 1991	Prospective	8600 PoM	The negative effect of parity on RF (OR, 0.68; 95% CI, 0.48-0.9)
Kauppi et al. 2011	Prospective	2028 (≥45 yrs)	≥ 3 births →↓ RF (OR, 0.50; 95% CI, 0.32-0.79)
Taylor et al. 2004	Prospective cohort	6787 (≥66 yrs)	Association of nulliparity with hip fracture (HR, 1.32; 95% CI, 1.11-1.57)
Michaelsson et al. 2001	Case-control	4640 (50-81 yrs)	Parity is modestly associated with reduced hip fracture (OR, 0.95; 95% CI, 0.9-1.0)
Specker et al. 2005	Cross-sectional	168 (40-80 yrs)	Association of High parity with increased bone size & strength
Huo et al. 2003	Case-control	156 (≥50 yrs)	Extended duration of breast feeding (≥ 24 months) is associated with reduced hip fracture (OR, 0.31; 95% CI, 0.15-0.64)
Cumming et al. 1993	Case-control	174 (≥65 yrs)	Lactation may protect against hip fracture [parous women OR, 1.53; 95% CI, 0.54-4.34)], [lactation OR, 0.64; 95% CI, 0.3-1.38)]
Boonyaratavej et al. 2001	Case-control	253(≥ 51 yrs)	Lactation (OR, 0.87; 95% CI, 0.8-0.94) is a protective factor against hip fracture
Naves et al. 2005	Prospective	255 (≥50 yrs)	Pregnancy is a protective factor against fracture (OR, 0.15; 95% CI, 0.03-0.62)
Mallmin et al. 1994	Case-control	367 (men & women) (40-80 yrs)	Increased RF in nilliparous women (OR, 1.8; 95% CI, 1.12-2.92)

RF = risk factor; PrM = premenopausal women; PoM = postmenopausal women; BMD = bone mineral density; yrs = years

PPA = postpartum amenorrhea; BMC = bone mineral content

Pregnancy, lactation and risk of fracture

Bone loss predisposes patients to bone fractures which may cause disabilities, and work loss and imposes high cost to the society. Based on the impact of pregnancy and lactation on bone mass, different effects can be seen. Some investigations revealed reduced risk of hip fracture due to parity (31, 81, 82). Kauppi et al confirmed the positive effect of parity on BMD and showed inverse association between risk of hip fracture and parity (83). The association of nulliparity with hip fracture was confirmed in several studies (84, 85). 

Michaëlsson et al. analyzed data from a population-based case-control study in Swedish women and reported 5% reduction of hip fracture per child which was influenced by use of oral contraceptives (OCP) (86). They observed that OCP increases the risk of hip fracture with no association between duration of lactation and risk of hip fracture. Also they found no correlation between body mass index (BMI), and duration of lactation with risk of fracture (86). Specker et al considered the effect of parity on bone size and strength as the factors which reduce risk of hip fracture (87).

Huo et al observed 13% reduced risk of hip fracture in association with every 6 months increase in lactation per child in Chinese women (88). In agreement with this study, Cumming et al and Kreiger et al observed the association of reduced risk of hip fracture with duration of lactation per child in a dose-dependent fashion (89, 90). In a case-control study in Thailand, addition of each child was associated with 13% reduction of risk of fracture (91) while some studies in Caucasians did not support it (31, 38, 86).

Naves et al. conducted a longitudinal study on Spanish women over 8 years and found pregnancy as an important protective factor for the incidence of Colles fractures (92). The results of the Mallmin et al study confirms this finding as they showed more Colles fractures in women who had never been pregnant (93). 

Pregnancy, lactation and bone biomarkers 

Because of the teratogenicity of X-ray on pregnant women, some investigators measure bone biomarkers as reliable indicators of bone status. Several studies demonstrated high maternal bone turnover specifically high levels of deoxypyridinoline (DpyD) and bone alkaline phosphatase (BALP) during pregnancy and 12 months postpartum in prospective studies (94, 95). Osteoprotegerin (OPG) which is a member of the tumor necrosis factor superfamily acts in counteraction with receptor activator of nuclear factor κB ligand (RANKL) and inhibits osteoclast activity. Production of OPG is induced by 17β-estradiol, increases over pregnancy and decreases during lactation (96, 97). It has been known that OPG is elevated in murine pregnancy which may protect maternal skeleton (98). Little is known about the role of OPG during pregnancy in human that might have placental origin. One study reported no significant change in OPG during pregnancy but increased level of OPG during labor (96)(Uemura et al., 2002). Naylor et al observed significant increase in OPG and β cross-linked C-telopeptide of type I collagen (β CTX) at 36 weeks of pregnancy followed by rapid postpartum decline (99). Their study showed no correlation between change in OPG and bone turnover or BMD (99). Vidal et al found OPG level of human milk 1000-fold higher than human serum. This high amount may prevent bone loss later in life (100). 

Holmberg-Marttila et al. assessed the postpartum changes in bone turnover markers and found significant postpartum decrease in β CTX (bone resorption marker) and increase in bone alkaline phosphatase (BALP), amino-terminal telopeptide of procollagen (PINP), osteocalcin (OC) (bone formation markers) as early as one month. They indicated the association of higher parity and longer history of lactation with lower bone turnover markers (45). 

Cross-sectional and longitudinal studies indicated 50% reduction of PTH during lactation (18, 49, 101-103). Also some studies reported decrease in procollagen I carboxypetides (PICP) in the first and second trimester and its increase in the last trimester as well as elevation of urine DpyD 2-3 fold during lactation higher than the third trimester (100-102, 104). 

In a longitudinal study, Chan et al compared BMD and bone biomarkers of lactating and non lactating Chinese mothers and reported significant decrement in BMD of lactating mothers in the first six months as returned to baseline at 12 months. Serum BALP was higher in lactating mothers and serum intact PTH (iPTH) increased in both groups (105). 

Carneiro et al reported higher levels of biochemical bone markers including CTX, N-terminal telopeptide (NTX), BALP, and osteocalcin in lactating mothers. They indicated the distinctive pattern of increased bone turnover in states of rapid bone loss (myeloma, cancer, etc) which displays uncoupling bone markers versus lactation and osteoblast-osteoclast coupling (106). Details of studies are summarized in Table 5.

**Table 5:** Studies which show the impact of parity and lactation on bone biomarkers

Author	Type of study	Subjects	Conclusion
**More et al. 2003**	**Prospective**	**20 pregnant women**	**↑bone markers during pregnancy and lactation; fail to reach baseline 12 months postpartum**
**Bezerra et al. 2002 **	**Cross-sectional**	**61 (14-19 yrs) & 77 (21-35 yrs)**	**Pregnancy and lactation affect bone turnover in adolescent and adults differently**
**Uemura et al 2002**	**-**	**14 (23-36 yrs)**	**Partial link between OPG and bone resorption after delivery**
**Naylor et al. 2003 **	**Longitudinal**	**17 (20-36 yrs)**	**No correlation between OPG change & bone turnover or BMD in pregnancy**
**Kovacs et al 1995 **		**33 lactating women**	**Lactation →↑PTHrP, Ca, P, ↓PTH**
**Cross et al 1995 **	**Longitudinal**	**10 women**	**↑Bone turnover during late pregnancy & lactation**
**Gallacher et al. 1994 **	**Longitudinal**	**10 pregnant women**	**↑PTHrP, BALP during pregnancy; ↑PTH postpartum**
**Chan et al 2005 **	**Longitudinal**	**23 postpartum**	**↑iPTH in lactating and non lactating mothers; ↑BALP in lactating mothers**
**Carneiro et al. 2010 **	**Prospective cohort**	**49 (24-41 yrs)**	**↑CTX, NTX, BALP, OC in lactating mothers than controls**

RF = risk factor; PrM = premenopausal women; PoM = postmenopausal women; BMD= bone mineral density; yrs = years

OPG = osteoprotegerin; Ca = calcium; PTHrP = PTH related protein; PTH = parathormone; P = Phosphorus

CTX = cross-linked C-telopeptide of type I collagen; BBALP= bone alkaline phosphatase; NTX = cross-linked N-telopeptide of procollagen

Oc = osteocalcin; iPTH = intact parathyroid hormone

## Discussion

In spite of controversial results of the mentioned studies, some investigators suggest that pregnancy causes bone loss and if pregnancy is followed by lactation, the bone density may return to normal level while the subjective reports of pregnancy-related osteoporosis and bone fractures in lactating mothers should be also taken into account (107, 108). Therefore, several contributory factors which may cause the discrepancies between results should be considered.

In order to have a more cautious and accurate conclusion, we should not ignore variations in the design of different studies (comparative groups, population characteristics, number of subjects, follow up period, and statistics), timing of the postpartum studies, nutritional status of mothers, racial differences in calcium homeostasis and bone metabolism, maternal age, parity, onset of menses, duration of lactation and bone sites which show inconsistencies of data^ (^109-111). Some studies conducted on premenopausal women while some performed on postmenopausal women and even pregnant women; as a result the wide variations between study subjects have influenced the outcomes. Retrospective studies relied on the past memory of the subjects about lactation period, lifestyle or physical activity which might be with mistakes. 

Actually, different bone sites, time and the method of bone densitometry may cause the discrepancy between results (12). Different studies show controversial results in different bone sites. Early studies showed advancing trabecular bone loss during pregnancy (112), however single and dual absorptimetry did not confirm former results (113, 114). More controversial results show increase in cortical bone density and decrease in trabecular bone density during pregnancy and postpartum (115, 116). It has been proposed that trabecular bone (lumbar spine) is more responsive to metabolic changes than the cortical bone (femoral neck, distal radius) (117). In addition, timing of bone loss in healthy women is different in bone sites. Trabecular bone mass lessens in every decade of life while cortical bone mass does not change in the third, fourth and fifth decades (118). 

The method of measuring bone density or bone metabolism may be another contributory factor. Dual-energy X-ray absorptimetry (DEXA) is the most common method of measuring bone density but because of its harmful effects on fetus, alternative methods may be used during pregnancy. Accordingly fewer studies using DEXA were performed or measured bone density and its changes during pregnancy. In the recent decade, quantitative ultrasonometry (QUS) has been used for determining bone density as a safer method in pregnant women and infants. Data obtained from QUS at the heel highly correlate with its BMD and biomechanical properties; some considered this method as sensitive as axial BMD in estimating vertebral and hip fracture (119, 120). This method is inexpensive, and radiation free for measuring bone density, bone quality, and risk of fracture (121). Several investigations into the issue have been conducted by measuring bone biomarkers but bone turnover markers do not show absolute changes in bone turnover however alteration in renal function during pregnancy and lactation as well as involution of the uterus affects bone turnover markers (45). Non-fasting state, and diurnal changes seem to influence CTX levels (45). Therefore, the influence of non bone tissue and its extent should not be ignored. 

Some investigators indicated the possibility of the contribution of body composition of mothers on bone mass. It has been determined that in adolescents, lean body mass and later in life, fat mass are predictors of bone mass, respectively (122). The relationship between bone loading and weight gain after delivery, increased calcium absorption during pregnancy, etc and their effects on bone mass was proposed (123) but there is no enough support for this hypothesis. 

There is the possibility of age-dependent contribution. Rate of bone metabolism in adolescents is higher than adults and this may lessen their sensitivity to adoption mechanisms in pregnancy and lactation (124). 

The effect of nutrition and well-defined life style should be considered. It is suggested that calcium intake can overcome the negative impact of lactation on bone mass (125 but studies show that bone loss due to lactation cannot be reversed by calcium supplementation (126); and vitamin D or PTH level is not related to bone mass change over lactation period (127). Some argue that maternal bone loss during lactation is a physiologic adaptation and cannot be prevented by calcium supplementation (128). Meanwhile, there are evidences that show decreased suckling decreases serum levels of prolactin and PTH which affects bone metabolism (127). 

In contrast, maternal PTH-related peptide could provide adequate calcium for infants by stimulating bone loss (101, 125) which is reversed after weaning (129). Variations in the time since last delivery and the average duration of breastfeeding per child may affect the results as well. 

There are reports which show recovery of BMD in the first 6-12 months after weaning (40). Hopkinson et al compared bone mineral content (BMC) in lactating and non lactating women during 2 years. They observed loss of 0.9% of BMC, 6 months after delivery which was recovered in 24 months whereas in non-lactating women, BMC increased 0.8% by 3 months postpartum and continued more rapidly in lactating mothers (12). 

Furthermore the importance of the results should be verified according to the comprehensive definition of lactation. Based on WHO definitions there are two types of breast feeding called exclusive or predominant. Exclusive breast feeding refers to absolute breast feeding for at least 4 months and if possible 6 months even no water included and predominant breast feeding refers to the breast milk as the main source of infants nourishment but the infant may get nourished with water, or juices. These definitions were not considered in the mentioned studies. In studies using questionnaire, the researcher/researchers should trust on the subjects’ memory even after 2-3 decades. 

The exact influence of hormonal status on bone during childbearing period is not fully known and its determination may be of great help. It has been suggested that during lactation, estrogen impose minor inhibitory effect on periosteal bone formation and permits periosteal expansion which increases bone size after weaning (130). 

Keeping above points in mind, it seems that pregnancy itself may lead to bone loss but if followed by lactation, it will have protective effect on bone density while the duration of lactation and parity may modulate its effect. Further investigation on this topic by considering the study limitations, contributory factors and using new safe techniques such as QUS is highly recommended.

## Confilict of Interests

Authors declare that they have no conflict of interest.
